# Supplementary material for: Intima-Media Thickness and Pulsatility Index of Common Carotid Arteries in Acute Ischaemic Stroke Patients with Diabetes Mellitus
Source: J Clin Med. 2022 Dec 29;12(1):246. doi: 10.3390/jcm12010246 (PMC9821495; doi:10.3390/jcm12010246)
Supplement: Supplementary file 1 [file jcm-12-00246-s001.zip › jcm-2079992-supplementary.pdf]

Supplementary Table S1. Univariable analysis (all variables shown).

Variable	Non Diabetics (N = 1.191)	Diabetics (N = 321)	Total (N = 1.512)	OR	OR - 95% CI	p-value
Age	73.20 (20.88)	74.80 (15.93)	73.79 (18.92)	1.02	1.01 - 1.03	<0.001
Sex (females)	527 / 1190 (44.3%)	121 / 320 (37.8%)	648 / 1510 (42.9%)	0.76	0.59 - 0.98	0.037
TOAST Mechanism						
Atherosclerotic	185 / 1150 (16.1%)	56 / 302 (18.5%)	241 / 1452 (16.6%)			
Cardioembolic	385 / 1150 (33.5%)	99 / 302 (32.8%)	484 / 1452 (33.3%)	0.85	0.59 - 1.24	
microangiopathic	116 / 1150 (10.1%)	35 / 302 (11.6%)	151 / 1452 (10.4%)	1.00	0.61 - 1.61	0.530
Other	48 / 1150 (4.2%)	8 / 302 (2.6%)	56 / 1452 (3.9%)	0.55	0.23 - 1.18	
Undetermined	416 / 1150 (36.2%)	104 / 302 (34.4%)	520 / 1452 (35.8%)	0.83	0.57 - 1.20	
Antiplatelets (ASA, Clopidogrel or Asasantine)	410 / 1180 (34.7%)	162 / 319 (50.8%)	572 / 1499 (38.2%)	1.94	1.51 - 2.49	<0.001
Anticoaculation	108 / 1180 (9.2%)	37 / 318 (11.6%)	145 / 1498 (9.7%)	1.31	0.87 - 1.92	0.193
Antihypertensives	670 / 1178 (56.9%)	241 / 318 (75.8%)	911 / 1496 (60.9%)	2.37	1.80 - 3.16	<0.001
Hypolipemic	301 / 1184 (25.4%)	143 / 320 (44.7%)	444 / 1504 (29.5%)	2.37	1.83 - 3.06	<0.001
Insulinotherapy	1 / 1178 (0.1%)	65 / 318 (20.4%)	66 / 1496 (4.4%)	NA	NA - NA	NA
Hypertension	833 / 1189 (70.1%)	274 / 320 (85.6%)	1107 / 1509 (73.4%)	2.55	1.84 - 3.60	<0.001
Hypercholesterolemia	894 / 1185 (75.4%)	271 / 319 (85.0%)	1165 / 1504 (77.5%)	1.84	1.33 - 2.59	<0.001
Smoking	279 / 1161 (24.0%)	78 / 316 (24.7%)	357 / 1477 (24.2%)	1.04	0.77 - 1.38	0.810
Atrial fibrillation	318 / 1186 (26.8%)	111 / 321 (34.6%)	429 / 1507 (28.5%)	1.44	1.11 - 1.87	0.007
Coronary disease	204 / 1180 (17.3%)	74 / 316 (23.4%)	278 / 1496 (18.6%)	1.46	1.08 - 1.97	0.015
Peripheral artery disease	67 / 1181 (5.7%)	41 / 317 (12.9%)	108 / 1498 (7.2%)	2.47	1.63 - 3.71	<0.001
Alcoholism	119 / 1181 (10.1%)	34 / 319 (10.7%)	153 / 1500 (10.2%)	1.06	0.70 - 1.58	0.762
Obstructive Apnea Syndrome	38 / 1123 (3.4%)	28 / 301 (9.3%)	66 / 1424 (4.6%)	2.93	1.75 - 4.84	<0.001
BMI	25.00 (6.00)	27.00 (6.25)	25.00 (5.00)	1.13	1.09 - 1.16	<0.001
NIHSS at admission	7.00 (11.00)	7.00 (11.00)	7.00 (11.00)	1.00	0.98 - 1.02	0.878
Reccurences						
none	952 / 1068 (89.1%)	247 / 279 (88.5%)	1199 / 1347 (89.0%)			
ischemic	107 / 1068 (10.0%)	31 / 279 (11.1%)	138 / 1347 (10.2%)	1.12	0.72 - 1.69	0.580
haemorrhage	9 / 1068 (0.8%)	1 / 279 (0.4%)	10 / 1347 (0.7%)	0.43	0.02 - 2.29	
Acute CTA /MRA significant Atherosclerosis	667 / 970 (68.8%)	194 / 232 (83.6%)	861 / 1202 (71.6%)	2.32	1.61 - 3.41	<.001
Acute CTA / MRA significant pathology in ischemic territory	613 / 969 (63.3%)	151 / 237 (63.7%)	764 / 1206 (63.3%)	1.02	0.76 - 1.37	0.897
Acute CTA / MRA stenosis or occlusion in extracranial vessels	264 / 960 (27.5%)	66 / 237 (27.8%)	330 / 1197 (27.6%)	1.02	0.74 - 1.39	0.915
IMT of R CCA	0.80 (0.30)	0.90 (0.40)	0.80 (0.30)	2.17	1.43 - 3.27	<0.001
IMT of L CCA	0.90 (0.40)	1.00 (0.40)	0.90 (0.40)	1.99	1.36 - 2.90	<0.001
PI of R CCA	1.56 (0.44)	1.64 (0.42)	1.58 (0.42)	1.76	1.27 - 2.44	0.001
RI of R CCA	0.76 (0.11)	0.78 (0.10)	0.77 (0.10)	14.05	3.17 - 64.06	<0.001
PI of LCCA	1.52 (0.45)	1.59 (0.36)	1.54 (0.44)	1.66	1.18 - 2.34	0.004
RI of R LCCA	0.76 (0.11)	0.77 (0.08)	0.76 (0.11)	11.56	2.58 - 53.73	0.001
PI of R ICA	1.23 (0.40)	1.33 (0.38)	1.25 (0.39)	2.11	1.46 - 3.06	<0.001
Ri of R ICA	0.68 (0.12)	0.71 (0.11)	0.68 (0.12)	27.47	[illegible].63 - 116.02	<0.001
PI of L ICA	1.23 (0.39)	1.31 (0.42)	1.25 (0.40)	2.15	1.52 - 3.04	<0.001
RI of L ICA	0.68 (0.12)	0.70 (0.12)	0.68 (0.12)	19.76	5.20 - 76.72	<0.001
heterogenous plaques R	301 / 330 (91.2%)	91 / 101 (90.1%)	392 / 431 (91.0%)	0.88	0.42 - 1.95	0.735
irregular plaques R	150 / 192 (78.1%)	56 / 69 (81.2%)	206 / 261 (78.9%)	1.21	0.62 - 2.49	0.593
erosion of plaques R	6 / 1191 (0.5%)	1 / 321 (0.3%)	7 / 1512 (0.5%)	0.62	0.03 - 3.63	0.637
ulceration of plaques R	0 / 1191 (0.0%)	0 / 321 (0.0%)	0 / 1512 (0.0%)	NA	NA - NA	NA
hyperechogenic plaques R	607 / 1191 (51.0%)	190 / 321 (59.2%)	797 / 1512 (52.7%)	1.40	1.09 - 1.79	0.009
hypoechogenic plaques R	90 / 1191 (7.6%)	26 / 321 (8.1%)	116 / 1512 (7.7%)	1.08	0.67 - 1.68	0.747
isoechogenic plaques R	533 / 1191 (44.8%)	166 / 321 (51.7%)	699 / 1512 (46.2%)	1.32	1.03 - 1.69	0.027
anechogenic plaques R	11 / 1191 (0.9%)	1 / 321 (0.3%)	12 / 1512 (0.8%)	0.34	0.02 - 1.73	0.225
acustic shadow of plaqu R	252 / 1191 (21.2%)	82 / 321 (25.5%)	334 / 1512 (22.1%)	1.28	0.96 - 1.70	0.097
heterogenous plaques L	280 / 299 (93.6%)	89 / 100 (89.0%)	369 / 399 (92.5%)	0.55	0.26 - 1.23	0.142
irregular plaques L	137 / 169 (81.1%)	56 / 67 (83.6%)	193 / 236 (81.8%)	1.19	0.57 - 2.62	0.649
erosion plaques L	0 / 1191 (0.0%)	1 / 321 (0.3%)	1 / 1512 (0.1%)	NA	NA - NA	NA
ulceration of plaques L	1 / 1191 (0.1%)	0 / 321 (0.0%)	1 / 1512 (0.1%)	NA	NA - NA	NA
hyperechogenic plaques L	551 / 1191 (46.3%)	191 / 321 (59.5%)	742 / 1512 (49.1%)	1.71	1.33 - 2.20	<0.001
hypoechogenic plaques L	74 / 1191 (6.2%)	33 / 321 (10.3%)	107 / 1512 (7.1%)	1.73	1.11 - 2.64	0.016
isoechogenic plaques L	490 / 1191 (41.1%)	158 / 321 (49.2%)	648 / 1512 (42.9%)	1.39	1.08 - 1.78	0.010
anechogenic plaques L	5 / 1191 (0.4%)	4 / 321 (1.2%)	9 / 1512 (0.6%)	2.99	0.74 - 11.37	0.119
acustic shadow of plaques L	253 / 1191 (21.2%)	68 / 321 (21.2%)	321 / 1512 (21.2%)	1.00	0.73 - 1.34	0.982

Supplementary Table S2. Predicted CCA PI average for patients with a specific characteristic (e.g., diabetic), keeping the other variables at their mean value.

IMT	Pred. CCA PI	Age	Pred. CCA PI	Sex	Pred. CCA PI
0.4	1.5556	50	1.4828	males	1.6154
0.6	1.5672	60	1.5323	females	1.5448
0.8	1.5787	70	1.5818		
1.0	1.5902	80	1.6313		
1.2	1.6017	90	1.6808		
**Lipid-lowering medication**	**Pred. CCA PI**	**DM**	**Pred. CCA PI**	**Smoking**	**Pred. CCA PI**
no	1.5633	no	1.5710	no	1.5953
yes	1.5991	yes	1.6374	yes	1.5532
**AF**	**Pred. CCA PI**	**BMI**	**Pred. CCA PI**	**NIHSS at admission**	**Pred. CCA PI**
no	1.5964	18	1.6191	5	1.5680
yes	1.5567	25	1.5881	10	1.5889
		30	1.5660	15	1.6099
		35	1.5438	20	1.6309
		40	1.5217	25	1.6519
**CTA Atherosclerosis**	**Pred. CCA PI**	**CTA significant precerebral stenosis**	**Pred. CCA PI**	**Side**	**Pred. CCA PI**
no	1.6226	no	1.5714	left	1.5641
yes	1.5703	yes	1.6210	right	1.6061
